# Fluency and reading comprehension as a performance indicator in the 3rd year of elementary school

**DOI:** 10.1590/2317-1782/20232021251en

**Published:** 2023-09-01

**Authors:** Vanessa de Oliveira Martins-Reis, Dâmaris Alves de Araújo Pedroso, Letícia Mendes de Almeida, Edlaine Souza Pereira, Luciana Mendonça Alves, Letícia Correa Celeste

**Affiliations:** 1 Faculdade de Ceilândia, Universidade de Brasília - UnB - Brasília (DF), Brasil.; 2 Programa de Pós-graduação em Ciências da Reabilitação, Universidade de Brasília - UnB - Brasília (DF), Brasil.; 3 Departamento de Fonoaudiologia, Faculdade de Medicina, Universidade Federal de Minas Gerais - UFMG - Belo Horizonte (MG), Brasil.; 4 Programa de Pós-graduação em Ciências Fonoaudiológicas, Faculdade de Medicina, Universidade Federal de Minas Gerais - UFMG - Belo Horizonte (MG), Brasil.

**Keywords:** Language, Reading, Educational Status, Students, Learning

## Abstract

**Purpose:**

to verify whether fluency and reading comprehension vary throughout the third year of elementary school and can be used as performance indicators in reading.

**Methods:**

this is a prospective longitudinal study. 53 children participated in the study, regardless of gender. Four assessments were carried out throughout the year, using the LEPIC software to record the reading made by the children, recording reading errors, as well as the answers to questions regarding comprehension.

**Results:**

there was an evolution of reading fluency and literal comprehension throughout the tests, but not in inferential comprehension.

**Conclusion:**

reading fluency and literal comprehension are good performance indicators in the third year of elementary school.

## INTRODUCTION

Reading is a complex activity with the aim of understanding what has been read^(1)^. This practice depends on the processing of visual, phonological, semantic, and linguistic stimuli and can also be associated with the ability to read smoothly, easily, and spontaneously without problems with word recognition^(1)^. According to a survey by the National Reading Panel (NRP)^(2)^, the fundamental skills and abilities for the literacy process are as follows: phonological awareness, familiarity with printed texts, metalanguage, phonemic awareness, knowledge of the alphabetic principle, decoding, fluency, and vocabulary. If a reader fails in any of these fundamental skills, there will be a decrease in speed, resulting in a lower reading fluency rate, which in turn may compromise understanding.

Speed, accuracy, and expressiveness are required for fluent reading^(3-5)^. Accuracy is the ability to decode accurately and can be measured by the number of words read correctly^(6)^. Speed is a characteristic of the development of automaticity^(6)^, which allows the reader to concentrate on understanding the text. Expressiveness characterizes how the reader interacts with the text and expresses their attitudes and emotions when reading the text^(7)^. Intermediate reading ability is established in the 3rd school year with the purpose of achieving automaticity so that reading becomes a pleasurable and motivating activity both in education and leisure, which provides greater demands on cognitive resources such as attention and memory.

Reading fluency is one of the skills that needs to be assessed throughout the literacy process, along with reading words and reading comprehension. To assess this ability, lists of words or texts can be used to obtain the number of words read correctly per minute^(1)^, which gives accurate and objective data on children's reading fluency. Reading texts aloud allows all components of reading fluency to be measured (accuracy, speed, and expressiveness).

Students who have difficulties with reading fluency tend to distance themselves from the knowledge gained by the habit of reading, which causes impairments in textual comprehension and content assimilation, which may well be an indicator of failure in their professional, social, and academic future^(5)^. This occurs because reading fluency and comprehension are strictly related, as fluid and effortless reading is an indicator of automaticity^(8)^, which allows the release of cognitive resources for comprehension activities^(9,10)^.

There are two types of processes involved in understanding: superior and basic^(11)^. Basic processes include decoding (which involves word recognition), understanding grammar rules, listening, and working memory. The superior processes include abilities to make inferences from a text, to have previous knowledge related to a text, develop cognitive strategies, and sensitivity to the structure of the text. They are composed of a series of abilities that help in understanding, such as working memory, vocabulary, and syntactic awareness^(11-13)^. In addition to cognitive-linguistic skills, other factors can interfere with understanding, such as motivational aspects, interests, and prior knowledge; social variables such as context and expectations; and attentional and memory resources, such as organization strategies and reasoning, that help to self-manage and achieve the reading objective^(14,15)^.

The organization of a text and its structure interfere with understanding, and its content needs to be properly processed and integrated into the knowledge acquired by the reader. Without prior knowledge that makes it possible to associate new information with pre-existing information, there is inadequate use of processes and strategies that result in comprehension problems^(16)^. Reading processes and strategies are improved with time, and the more time an individual spends reading, the more they develop their comprehension abilities.

Comprehension can be assessed both during and after reading. In the after-reading assessment retelling, answering open-ended and closed-ended questions and problem-solving can be used. During reading assessment, there are the following: reading time, lexical decision tasks, naming, and recognition. Multiple-choice questions are a technique that is considered to be practical, fast, objective, and efficient since it allows for an accurate assessment of understanding without interference from the evaluator's subjectivity^(3)^. To answer questions, the information in the text must be integrated with their prior knowledge, experiences, relationships of ideas within or between literal and inferential sentences, and connecting thoughts to complement information that is not explicit^(17)^.

An adequate teaching program must include a set of skills and competencies described in the NRP^(2)^, including fluency and reading comprehension so that there are no pedagogical implications. In addition, it is essential to monitor these skills throughout the school years so that necessary adjustments can be made to strengthen a child's learning; however, this is not the reality of public education policies or pedagogical practice, especially for reading fluency.

The 2019 National Literacy Policy (PNA) is a state policy aimed at literacy based on scientific evidence and relates to some of the aims of the 2014 National Education Plan, specifically that all children should be literate by the end of the 3rd year of elementary school (EF) and there should be a general increase in literacy rates. The PNA clearly addresses the literacy process, citing the fundamental skills and abilities of the NRP^(2)^ and those involved in the process. In addition, it emphasizes the importance of evaluating and monitoring the public policy itself, recognizing that there is a deficiency in this aspect, but without pointing out what the evaluation procedures are. The document, however, encourages "the development of indicators to assess school effectiveness in literacy”, although it does not address how the teacher should assess reading fluency and only points out its importance and presents a table with reference values for the number of words per minute for each school year, with 90 words per minute being the average established for the third year of EF.

Data from the National Literacy Assessment carried out in 2016, and from the Basic Education Assessment System (Saeb), carried out in 2019 show progress in the reading performance of children in the early years of Elementary School, but there is a discrepancy in performance when comparing public and private schools. To improve the quality of learning, the Ministry of Education and Culture created vacancies for a Literacy Practices course in 2020. The course is based on the PNA and is aimed at teachers and other professionals involved in the literacy process, with teaching strategies, activities and assessments aimed at the 1st and 2nd years of Elementary School. Reading fluency is addressed in module 4 of the course, with strategies to develop speed, accuracy, and prosody. However, in addition to learning strategies, it is important to monitor students' progress in reading fluency, as this allows the teacher to know in greater detail the reading problems of each student and review pedagogical planning. The NRP^(2)^ suggests that formal assessments should take place regularly at bimonthly intervals, for example, and informally through teacher observations in the classroom and other small tests and oral tests. The PNA addresses reading fluency and reinforces its importance but does not propose a practical practice for objective assessment of reading fluency. In addition, no studies were found in Brazil that carried out longitudinal monitoring of this skill throughout the school year, and in many other countries such studies are still not commonplace.

The objective assessment of reading fluency when done manually requires time for professionals, the alternative is to use *Lepic* ^(18)^ software which has proven to be a valid and feasible tool for assessment and monitoring that can be used by any previously trained person, and may be a viable alternative to use in education, especially where there are many students to evaluate.

As previously mentioned, it is essential that fluency and reading comprehension are monitored during schooling using scientifically validated instruments and measures to guide teachers. This study aimed to verify whether fluency and reading comprehension vary throughout the school year and can be used as an indicator of a student's performance throughout the third year of Elementary School. This year of schooling was chosen due to the target established before the changes made by the National Common Curricular Base (BNCC), which recommended that children should be literate by the end of the 3rd year of Elementary School, with a period of two years for its implementation. In this sample, monitoring was carried out over a year with emphasis on the performance of these skills. Development of performance is expected both in fluency by reaching 90 words per minute, and in reading comprehension by the number of individual questions answered correctly. The study may contribute to the work of educational speech therapists by way of implementing the monitoring of fluency and reading comprehension in Elementary Schools.

## METHODS

This is a prospective longitudinal observational study, approved by the Research Ethics Committee of the institution in accordance with No. 2,499,005. School and sample selection was done at convenience. Parents or guardians signed a consent form (TCLE) and the children signed a term of agreement for minors before starting data collection.

### Participants

All students enrolled in the 3^rd^ year of EF in early 2019 were eligible to participate in the study. 171 consent forms were sent to children and their parents/guardians enrolled in the 3rd year of a public school, 90 from the morning classes and 81 from the afternoon classes. Only 55 forms were signed and returned, 31 from students attending morning classes, and 24 from afternoon classes. The sample size was 53 children from the 3rd year of elementary school. As an inclusion criterion, children had to be enrolled in the third year of elementary school and be considered literate by the responsible teacher. Children with uncorrected visual and auditory problems or with neurological, psychiatric, and communicative alterations, as well as those with failures in the decoding of isolated words were excluded. Exclusion criteria were determined through a parental questionnaire and by reading a list of words and pseudowords.

### Instruments

To assess fluency and reading comprehension, a reading passage entitled “A Coisa” (The Thing) as adapted by Salles and Parente^(13)^ was selected. *Lepic* software was used to carry out the evaluation. This program aims to semi-automatically and instantly assess reading fluency, enabling evaluation, diagnosis and monitoring^(18)^. The analysis began by recording the children reading the text, which had approximately 200 words, followed by the completion of the text comprehension questionnaire. The questionnaire had 10 questions, 5 of which were literal and 5 inferential. The audio was recorded by the software itself. The program registers reading errors, words read repeatedly, and words inserted or not read. After the registration process, *Lepic* can generate individual and group reports.

### Procedures

Children were recruited at school during lesson times and were accompanied by the researchers to a room made available by the school principal on the day of the evaluation. Each student was asked to read the text "A Coisa” (The Thing)^(13)^ silently, taking as much time was as necessary. Afterwards, they were asked to read the same text aloud and were told that the reading would be recorded by the software. After the reading, the children answered the questionnaire, and they were not allowed to consult the text to help them answer the questions. The questions were read aloud by the researchers, as well as the respective answer options, and the child could follow the reading of the questionnaire on a computer screen. At the end of the procedure, which took on average 15 minutes, the child was sent back to the classroom.

We chose to monitor fluency and reading comprehension during the student's bimonthly evaluations, which took place in April, June, September, and November. If a child was absent on the day of the assessment, the information was excluded.

### Data analysis

*Lepic* performed the analysis of the total reading time with 90 words per minute (WPM) being used in the research^(18)^, resulting in a performance analysis by child, class and school. To calculate the reading fluency parameter, the number of words read per minute is divided by the total time in seconds. To analyze reading comprehension, the total number of correct answers of the literal and inferential questions and the total of comprehension were calculated.

After the four data collections, the number of words per minute, total reading time and performance in the comprehension task were extracted from the software. The data obtained was entered into an Excel spreadsheet for checking and processing. Measures of central tendency and dispersion were calculated for each of the studied variables. To verify the development of performance in fluency and reading comprehension throughout the tests, the Wilcoxon test was used with a significance level of 5%. For statistical analysis, SPSS software version 21.0 was used.

## RESULTS

Tables 1 and 2 show the results of the fluency and reading comprehension from the four annual tests. The improvement in the reading fluency parameter can be seen mainly in tests three and four (Table 1). We also found that in the second test the values decreased slightly, which may be due to the sample size. The intragroup variability is notable, given the high values of the standard deviation and the difference between the minimum and maximum values. Table 2 shows statistically significant differences in all the comparisons carried out.

**Table 1:** Descriptive measures of fluency and reading comprehension throughout the four tests

	**FLUENCY**			
	**WPM1 (n=42)**	**WPM2 (n=18)**	**WPM3 (n=43)**	**WPM4 (n=28)**
Average	67	65.06	84.12	91.75
Median	69.5	66.5	86	86.5
Standard deviation	25.9	26.47	26.46	29.1
Minimum	22	26	37	35
Maximum	123	114	134	139
	**COMPREHENSION**			
	**Literal1 (n=40)**	**Literal2 (n=19)**	**Literal3 (n=33)**	**Literal4 (n=35)**
Average	3.85	4	4.55	4.54
Median	4	5	5	5
Standard deviation	1.25	1.37	0.87	0.74
Minimum	1	1	1	3
Maximum	5	5	5	5
	**Inferential1 (n=40)**	**Inferential2 (n=19)**	**Inferential3 (n=33)**	**Inferential4 (n=35)**
Average	3.95	3.58	4.12	3.8
Median	4	4	4	4
Standard deviation	1.06	1.17	0.96	1.18
Minimum	1	1	0	0
Maximum	5	5	5	5
	**Total1 (n=40)**	**Total2 (n=19)**	**Total3 (n=33)**	**Total4 (n=35)**
Average	7.8	7.58	8.67	8.37
Median	8	8	9	9
Standard deviation	1.9	2.27	1.63	1.73
Minimum	2	3	1	3
Maximum	10	10	10	10

**Caption:** WPM= words per minute

**Table 2:** Result of the Wilcoxon test for the comparison of fluency and reading speed during the tests

		**TESTED**					
		**1ª x 2ª**	**1ª x 3ª**	**1ª x 4ª**	**2ª x 3ª**	**2ª x 4ª**	**3ª x 4ª**
WPM	p-value	0.002*	<0.001*	<0.001*	0.003*	0.008*	0.001*
Literal comprehension	p-value	0.885	0.009*	0.007*	0.443	0.131	0.608
Inferencial comprehension	p-value	0.317	0.206	0.774	0.257	0.317	0.206
Total comprehension	p-value	0.317	0.013*	0.05*	0.268	0.279	0.917

* p<0.05

**Caption:** WPM= words por minuto; Wilcoxon test

The questionnaires with answers to the literal questions which were completed after the text was read showed an improvement as can be seen in Table 1, mainly in tests three and four. There was a statistically significant improvement when comparing the first test with the third and fourth tests (Table 2).

No significant improvement was seen in the children's performance in the inferential questions of the questionnaire, and we saw some difficulties when compared to literal questions (Table 1). However, there was an improvement in test three, but this improvement was not significant (Table 2).

The total performance in reading comprehension in the four tests varied significantly with each test and showed an improvement when making a comparison, which is to be expected due to a good performance in the literal questions (Table 1). We also found a decrease in performance from the first to the second test, as well as from the third to the fourth test. Table 2 shows that there is a statistically significant increase when comparing the first test with the third and fourth tests.

## DISCUSSION

The object of this study was to assess whether fluency and reading comprehension vary throughout the 3rd year of Elementary School. The findings of the study indicated that there was an improvement in fluency and global reading comprehension throughout the 3rd school year of Elementary School, which was objectively and semiautomatically measured by electronic means. The assessment of reading fluency and comprehension was carried out following a theoretical framework already established in the area^(1,4,8,19-21)^, and the results showed a measurable, objective, and gradual development (Tables 1 and 2). Such findings allow us to highlight the importance of including fluency and reading comprehension monitoring throughout the school years as an indicator of performance. With these results, the pedagogical team can outline the necessary adjustments in planning to improve the students' performance both in fluency and in reading comprehension.

According to the results, reading fluency is variable in the same school year, being considered a practical indicator of quality, not only for an accurate evaluation of the students' performance, but also to accompany them throughout the year and as they advance through school graded, which supports other studies^(20,21)^.

Some other factors can help to improve reading fluency, such as shorter words, which tend to be decoded more easily, as well as those with a simpler syntactic structure^(12)^ and the number of pauses during the reading of a text^(4)^. A study carried out with 97 students from the 3rd, 4th and 5th grades^(4)^ showed a relationship between the number of breaks and the reading rate for 3rd grade students. These students had more pauses and difficulties in decoding, as well as a slower reading rate and difficulties in understanding. This data suggests that a possible consolidation of reading in the 3rd year is dependent on the learning ability of each student. Consequently, this analysis becomes relevant because in our sample reading fluency in the 3rd year was not yet established but was in the process of development.

Of the results found in this study of the four reading speed tests only the last evaluation had an average compatible with that recommended by the PNA (90 WPM), which are values referred to in national public policies. This target was reached only at the end of the year, after reading practice in different subjects, which stimulated common literacy skills in different tasks. About 50% of the students did not achieve the target, which confirms the need to have mechanisms in place for monitoring reading fluency in schools, not only regular evaluations, but also to verify reading fluency during the year so that there is a clear picture of what is happening within the parameters of reading fluency and allowing for strategies to be drawn up.

In reference to understanding the literal questions there were no two months’ development by the students, however there was an improvement when comparing the first with the third and fourth tests. As for inferential understanding no improvement was seen throughout the school year. In another study whose objective was to evaluate the comprehension of a text in children of the second and third year of elementary school, a questionnaire was answered based on the reading of the text "A Coisa", the same text used in our study. Literal questions are identified as questions related to memory, calling them memory events^(13)^. In the results of the study, the performance for answering literal questions was slightly higher than inferential questions, which is consistent with the findings in our study.

Cunha and Capellini^(17)^ also found a lower performance regarding inferential questions in their results, and greater difficulty on the part of the students, since very often there is not enough prior knowledge to link ideas, which also endorses our study. These findings reinforce the need for systematic and explicit work in the initial elementary school series with a focus on reading comprehension, in addition to the other skills identified in the NRP, mainly in vocabulary^(22)^. Readers who are more informed about a topic can have more inferences and better reasoning and more coherent representations, resulting in better understanding, identification of meanings and the ability to relate the text presented from prior knowledge^(23)^. The children's difficulty with inferential questions alone does not justify how this parameter was developed. Analysis of the school curriculum and an in-depth study of the textual discussions held in the classroom can help to better understand this issue.

Other theories about comprehension difficulties may be due to extrinsic or intrinsic factors. Intrinsic factors such as linguistic skills related to the ability to make inferences, and cognitive skills related to prior knowledge stand out most. Extrinsic factors on the other hand, can be due to socioeconomic factors, poor health, frequent absences and an inadequate school environment^(24)^. The school where the research was carried out is located in a region of greater social vulnerability which may have influenced the performance of the children. The 2018 report by Codeplan (Department of finance, planning, budget and management of the Federal District) pointed out that the school in which the research was carried out is located in the administrative region of Samambaia of the Federal District, which is part of the western planning unit along with Taguatinga, Sol Nascente, Ceilândia and Brazlândia, these being the most populous region of DF. Most people who declare themselves as students aged between 4- and 24-years old attend public school. In the age group studied aged between 6 and 14 years old in the GDF report, 97% attend school, and this figure decreases with an increase in age, for example (86% between 15 and 17 years old). 35.7% of the population aged 25 or over had completed high school, 22.7% had not completed elementary school and 4.7% had no schooling at all. The participation rate for Samambaia was 63.7% in the data collection period (2018). Some other statistics worth noting are that 35.5% of the population aged between 18 and 29 years old neither work nor study. Only 62.8% of workers reported having a formal employment contract. 70.9% of the population has a gross income of up to 2 Brazilian minimum salaries^(25)^.

Another finding of this research concerns the high intragroup variability (Table 1). The high value of standard deviation in reading fluency patterns was also seen in other studies on the subject in Brazil^(26,27)^. Such findings show that during initial literacy, students develop at a different pace, and it is important for educators to understand the differences and invest in reading fluency.

Development of knowledge is the best way to improve reading comprehension^(14)^. This process should take place during the first school grades through listening, talking and activities, as well as through reading itself.

In this study, it was decided to monitor fluency and reading comprehension every two months, coinciding with other evaluations carried out by teachers at the school. International studies carry out evaluations every four months^(20,21)^. The results found, that when assessing reading comprehension that three assessments per year are sufficient. In light of this it is suggested that educational speech-language pathologists together with a pedagogical team should carry out assessments at the beginning, in the middle and at the end of the school year utilizing validated instruments.

The limitations of the study were the sample size and the difficulties of testing all the students in all the stages due to absences of the students on the day of collection, or because they refused to read aloud when asked, or because teachers did not give permission for them to leave the classroom during lesson times. Their availability was also affected by various events that occurred constantly at the school, or even cancellation of classes, without the researchers being informed in advance.

According to Arnesen et al.^(20)^, this loss of data is to be expected due to student absences, and unforeseen events that can happen during a prospective longitudinal study. Absences can occur not only on the part of the student, but also on the part of the teacher, for health reasons, and students may also be transferred to another school, which we also found.

Using the same text in the 4 evaluations of this research did not prove to be a problem or limiting factor since we found recurring difficulties in some students. Furthermore, according to a meta-analysis improvement in fluency is only achieved by more than 4 repetitions of the same text just before the assessment^(8,28)^.

## CONCLUSION

Fluency and reading comprehension varied throughout the school year in children attending the 3rd year of Elementary School, and this can be used as an indicator of reading performance. From the results found in our study and international studies, it is suggested that screening should be carried out at the beginning of the school year and monitoring in the middle and at the end of the school year, which would allow for early intervention and help to reduce failure rates. Monitoring becomes easier and more accurate by using *LEPIC* software because as well as being easy to use, the evaluation by the software is quick and automatic and provides both individual and collective data, allowing for the identification of students who require changes of teaching strategies.

*LEPIC* proved to be an excellent tool for screening and monitoring, furthermore it can be used by anyone, including the teachers themselves, as long as they have had prior training.
