# Supplementary material for: Fluency and reading comprehension as a performance indicator in the 3rd year of elementary school
Source: Codas. 2023 Sep 1;35(6):e20210251. doi: 10.1590/2317-1782/20232021251en (PMC10547134; doi:10.1590/2317-1782/20232021251en)
Supplement: Translation

# Translation: A fluência e compreensão leitora como indicador de desempenho no 3^o^ ano do Ensino Fundamental

**DOI:** 10.1590/2317-1782/20232021251pt

**Authors:** Vanessa de Oliveira Martins-Reis, Dâmaris Alves de Araújo Pedroso, Letícia Mendes de Almeida, Edlaine Souza Pereira, Luciana Mendonça Alves, Letícia Correa Celeste

**Affiliations:** 1 Faculdade de Ceilândia, Universidade de Brasília - UnB - Brasília (DF), Brasil.; 2 Programa de Pós-graduação em Ciências da Reabilitação, Universidade de Brasília - UnB - Brasília (DF), Brasil.; 3 Departamento de Fonoaudiologia, Faculdade de Medicina, Universidade Federal de Minas Gerais - UFMG - Belo Horizonte (MG), Brasil.; 4 Programa de Pós-graduação em Ciências Fonoaudiológicas, Faculdade de Medicina, Universidade Federal de Minas Gerais - UFMG - Belo Horizonte (MG), Brasil.

**Keywords:** Linguagem, Leitura, Escolaridade, Estudantes, Aprendizagem

## Abstract

**Objetivo:**

verificar se a fluência e a compreensão leitora variam ao longo do terceiro ano do Ensino Fundamental, podendo ser utilizados como indicadores de desempenho em leitura.

**Método:**

trata-se de estudo longitudinal prospectivo. Participaram do estudo 53 crianças, sem distinção de sexo. Foram realizadas quatro avaliações ao longo do ano, utilizando-se do *software LEPIC* para gravar a leitura feita pelas crianças, registrar os erros de leitura, bem como as respostas às questões referentes à compreensão.

**Resultados:**

observou-se evolução da fluência de leitura e na compreensão literal ao longo das testagens, mas não na compreensão inferencial.

**Conclusão:**

a fluência de leitura e a compreensão literal apresentam-se como bons indicadores de desempenho no terceiro ano do Ensino Fundamental.

## INTRODUÇÃO

A leitura é uma atividade complexa que tem como objetivo a compreensão do que foi lido^(1)^. Essa prática depende do processamento de estímulos visuais, fonológicos, semânticos e linguísticos e pode ser associada também a habilidade de ler de forma suave, fácil e espontânea sem a ocorrência de problemas no reconhecimento das palavras^(1)^. Segundo levantamento do National Reading Panel (NRP)^(2)^, as competências e habilidades fundamentais para o processo de alfabetização são: consciência fonológica, familiaridade com textos impressos, metalinguagem, consciência fonêmica, conhecimento do princípio alfabético, decodificação, fluência e vocabulário. Assim, se o leitor apresentar falha em alguma competência e habilidade fundamental, há uma queda de velocidade, logo uma menor taxa de fluência de leitura, que por sua vez, poderá comprometer a compreensão.

Para a leitura ser considerada fluente é necessário velocidade, acurácia e expressividade^(3-5)^. A acurácia é a habilidade de decodificar de forma precisa, podendo ser mensurada por meio do número de palavras lidas corretamente^(6)^. A velocidade é uma característica de desenvolvimento da automaticidade^(6)^, o que permite ao leitor se concentrar na compreensão do texto. A expressividade caracteriza como o leitor interage com o texto e expressa suas atitudes e emoções ao ler o texto^(7)^. A considerada leitura intermediária estabelece-se no 3º ano escolar com finalidade de alcançar a automatização de modo que a leitura se torne uma atividade de contentamento e motivação tanto no sentido de lazer quanto no educacional, o que proporciona maiores demandas de recursos cognitivos, como atenção e memória.

Portanto, a fluência leitora é uma das habilidades que precisam ser avaliadas ao longo do processo de alfabetização, juntamente com a leitura de palavras e compreensão de leitura. Para avaliar essa competência podem ser utilizadas lista de palavras ou textos para obtenção do número de palavras lidas corretamente por minuto^(1)^, gerando um dado preciso e objetivo sobre a fluência de leitura das crianças. A leitura de textos em voz alta permite que sejam observados todos os componentes da fluência leitora (precisão, velocidade e expressividade).

Escolares que apresentam dificuldades com fluência de leitura distanciam-se dos conhecimentos fornecidos pelo hábito de ler, acarretando prejuízos na compreensão textual e assimilação de conteúdo, podendo ser indicador de insucesso no futuro profissional, social e acadêmico^(5)^. Isso ocorre porque a fluência leitora e a compreensão estão estritamente relacionadas, pois uma leitura fluida e sem esforço é um indicador de automaticidade^(8)^, o que permite liberação de recursos cognitivos para a atividade de compreensão^(9,10)^.

Existem dois tipos de processos envolvidos na compreensão, os superiores e os básicos^(11)^. Os processos básicos incluem a decodificação (que implica no reconhecimento das palavras), a compreensão das regras gramaticais, a compreensão oral e a memória de trabalho. Já nos processos superiores estão as habilidades de realizar inferências a partir de um texto, possuir conhecimentos prévios relacionados ao texto, estratégias cognitivas desenvolvidas e sensibilidade à estrutura do texto, compondo uma série de habilidades que auxiliam na compreensão, como memória de trabalho, vocabulário e consciência sintática^(11-13)^. Além das habilidades cognitivo-linguísticas, outros fatores podem interferir na compreensão, como aspectos motivacionais, interesses e conhecimento prévio, variáveis sociais como contexto e expectativas, recursos atencionais e de memória, tais como estratégias de organização e realização de raciocínios que ajudam a autogerenciar e alcançar o objetivo de leitura^(14,15)^.

A organização do texto e sua estrutura interferem na compreensão e seu conteúdo precisa ser adequadamente processado e integrado nos conhecimentos adquiridos pelo leitor. Dessa forma, na ausência de conhecimentos prévios que possibilitam associar informações novas à informação pré-existente, há uso inadequado de processos e estratégias que resultam em problemas de compreensão^(16)^. Os processos e estratégias de leitura vão sendo aprimorados ao longo da trajetória como leitor, ou seja, quanto mais o indivíduo pratica a leitura, mais a habilidade de compreensão é desenvolvida.

A compreensão pode ser avaliada durante e após a leitura. Na avaliação após a leitura podem ser utilizados o reconto, resposta a questões abertas e fechadas e resolução de problemas. Quanto à avaliação durante a leitura, temos o tempo de leitura, tarefas de decisão lexical, nomeação e reconhecimento. As questões de múltipla escolha são uma técnica considerada prática, rápida, objetiva e eficiente por possibilitar uma avaliação precisa da compreensão sem sofrer interferências da subjetividade do avaliador^(3)^. Assim, para responder às questões, deve ocorrer a integração da informação do texto aos seus conhecimentos prévios, experiências, a relação das ideias dentro ou entre sentenças literais e inferenciais, conectando pensamentos para complementar informações que não estão explícitas^(17)^.

Um programa adequado de ensino deve incluir o conjunto das habilidades e competências descritas no NRP^(2)^, incluindo a fluência e a compreensão leitora, para que assim não haja implicações pedagógicas. Além disso, é fundamental o monitoramento dessas habilidades ao longo do ano letivo para que os ajustes necessários sejam realizados a fim de potencializar o aprendizado das crianças. Entretanto, não é o que se observa nas políticas públicas de educação e na prática pedagógica, em especial para a fluência leitora.

A Política Nacional de Alfabetização (PNA) de 2019 é uma política de Estado voltada para a alfabetização, embasada em evidências científicas, relacionando-se com algumas metas do Plano Nacional de Educação de 2014, especificamente a alfabetização de todas as crianças até o final do 3^o^ ano do Ensino Fundamental (EF) e ao aumento da taxa de alfabetização da população. A PNA aborda com clareza o processo de alfabetização, citando as competências e habilidades fundamentais do NRP^(2)^ e os agentes envolvidos no processo. Além disso, ressalta a importância de realizar avaliação e monitoramento da política pública em si, reconhecendo que há uma deficiência nesse aspecto, mas sem apontar quais são os procedimentos de avaliação. O documento, contudo, estimula "o desenvolvimento de indicadores para avaliar a eficácia escolar na alfabetização". Apesar de não abordar como o professor deve avaliar a fluência de leitura, aponta sua importância e apresenta uma tabela com valores de referência para o número de palavras por minuto para cada ano escolar, sendo 90 palavras por minuto a média estabelecida para o terceiro ano do EF.

Dados da Avaliação Nacional de Alfabetização, realizada em 2016, e do Sistema de Avaliação da Educação Básica (Saeb), realizada em 2019, mostram um avanço no desempenho em leitura das crianças dos anos iniciais do Ensino Fundamental, mas há uma discrepância de desempenho ao se comparar escolas públicas e privadas. Com intuito de melhorar a qualidade da aprendizagem, o Ministério da Educação e Cultura abriu vagas para o curso de Práticas de Alfabetização em 2020. O curso baseia-se na PNA e é voltado para professores, e outros profissionais envolvidos no processo de alfabetização, com estratégias de ensino, atividades e avaliações voltadas para o 1o e 2o ano do Ensino Fundamental. A fluência leitora é abordada no módulo 4 do curso, trazendo estratégias para desenvolver velocidade, precisão e prosódia. Entretanto, além de estratégias de aprendizado, é importante realizar o monitoramento do progresso dos alunos na fluência de leitura, pois isso permite ao professor conhecer, com mais riqueza de detalhes, os problemas de leitura de cada aluno e rever o planejamento pedagógico. O NRP^(2)^ sugere que a avaliação aconteça regularmente tanto de modo formal, por meio de provas bimestrais, por exemplo, como de modo informal através de observações do professor na sala de aula, pequenos testes e provas orais. A PNA aborda a fluência de leitura e reforça sua importância, mas não propõe uma prática exequível quanto à avaliação objetiva da fluência leitora. Além disso, não foram encontrados estudos no Brasil que realizem acompanhamento longitudinal dessa habilidade ao longo do ano letivo, e em outros países, estudos com tal proposta ainda não são frequentes.

A avaliação da fluência leitora de forma objetiva, quando feita manualmente, pode demandar um tempo por parte dos profissionais, por isso, o uso do *software Lepic* ^(18)^ tem se mostrado uma ferramenta válida e exequível de avaliação e monitoramento que pode ser feita por qualquer pessoa previamente treinada, podendo ser uma alternativa para uso no contexto educacional, onde há muitos estudantes para avaliação.

A partir do exposto, observa-se que é fundamental que a fluência e a compreensão leitora sejam monitoradas ao longo do ano letivo por meio de instrumentos e medidas validados cientificamente para nortear o trabalho do professor. Dessa forma, o estudo aqui proposto teve como objetivo verificar se a fluência e a compreensão leitora variam ao longo do ano letivo, podendo ser utilizadas como indicadores de desempenho do escolar ao longo do terceiro ano do EF. A escolha desse ano escolar se deve à meta estabelecida antes das mudanças realizadas pela Base Nacional Comum Curricular (BNCC), que preconizavam que a criança deveria ser alfabetizada até o final do 3º ano do EF, com prazo de dois anos para sua aplicação. Foi realizado o monitoramento ao longo de um ano nessa amostra com ênfase no desempenho dessas habilidades. É esperada evolução no desempenho tanto na fluência, através do alcance de 90 palavras por minuto, como na compreensão leitora, por meio da evolução da taxa de acerto das questões, individualmente. O estudo poderá contribuir para o trabalho do Fonoaudiólogo Educacional, no sentido de implementar o monitoramento da fluência e da compreensão leitora no Ensino Fundamental I.

## MÉTODO

Trata-se de estudo observacional longitudinal prospectivo, aprovado pelo Comitê de Ética em Pesquisa da instituição conforme o parecer Nº 2.499.005. A seleção da escola e da amostra foi feita por conveniência. Os pais ou responsáveis assinaram o Termo de Consentimento Livre e Esclarecido (TCLE) e as crianças assinaram um Termo de Assentimento do menor antes de se iniciar a coleta de dados.

### Participantes

Todos os estudantes matriculados no 3º ano do EF no início de 2019 eram elegíveis a participar do estudo. Foram enviados 171 termos de consentimento para as crianças e seus pais/responsáveis matriculadas no 3º ano de uma escola de financiamento público, sendo 90 do turno da manhã e 81 do turno da tarde. Apenas 55 termos retornaram assinados, sendo 31 de alunos do turno da manhã, e 24 do turno da tarde. O total da amostra é de 53 crianças do 3º ano do EF. Como critério de inclusão, a criança precisava estar matriculada no terceiro ano do EF e ser considerada alfabetizada pelo professor responsável. Foram excluídas crianças com problemas visuais e auditivos não corrigidos e com alterações neurológicas, psiquiátricas e comunicativas, bem como as que apresentaram falhas na decodificação de palavras isoladas. Os critérios de exclusão foram levantados por meio de questionário aos pais e da leitura de uma lista de palavras e pseudopalavras.

### Instrumentos

Para avaliação da fluência e da compreensão leitora foi utilizado o texto “A coisa” adaptado por Salles e Parente^(13)^. Para realizar a avaliação, foi utilizado o *software Lepic,* que possui como objetivo avaliar de forma semiautomática e instantânea a fluência leitora, possibilitando avaliação, diagnóstico e monitoramento^(18)^. Essa análise é iniciada a partir da gravação da leitura oral do texto, que apresenta aproximadamente 200 palavras, seguido por aplicação do questionário de compreensão do texto, composto por 10 questões, sendo 5 literais e 5 inferenciais. O áudio foi gravado pelo próprio software. No programa é possível registrar o erro de leitura, palavras lidas repetidamente e palavras inseridas ou não lidas. Após os registros, o *Lepic* é capaz de gerar relatórios individuais e por grupos.

### Procedimentos

Inicialmente as crianças foram recrutadas, durante o período de aula, e acompanhadas pelas pesquisadoras a uma sala disponível pela direção no dia da avaliação. Cada escolar era orientado a realizar a leitura do texto "A coisa"^(13)^ de forma silenciosa, levando o tempo que fosse necessário. Em seguida, era solicitada a leitura do mesmo texto em voz alta, sendo informado que a leitura em voz alta seria gravada pelo *software Lepic*. Após a leitura em voz alta, foi aplicado o questionário de compreensão de texto, no qual a criança não podia fazer a consulta ao texto para responder. As perguntas foram lidas em voz alta pelas pesquisadoras, assim como as respectivas opções de resposta, à medida que o avaliado acompanhava a leitura do questionário pela tela do computador. Ao término do procedimento, que levou em média 15 minutos, a criança foi encaminhada de volta à sala de aula.

Optou-se por realizar o monitoramento da fluência e da compreensão leitora no período de avaliações bimestrais do escolar, que aconteceram nos meses de abril, junho, setembro e novembro. Dessa forma, caso a criança não estivesse presente no dia da avaliação, a informação era perdida.

### Análise dos dados

O software *Lepic* realiza a análise a partir do tempo total de leitura, sendo utilizado na pesquisa palavras por minuto (PPM)^(18)^. Ao final da avaliação, é apresentada análise com desempenho individual, da classe e da escola. Para obtenção de parâmetro da fluência de leitura, calcula-se o número de palavras lidas por minuto dividido pelo tempo total em segundos. Para analisar a compreensão de leitura, considerou-se o total de acertos apresentado pelo escolar nas questões literais, inferenciais e o total na compreensão.

Após realização das quatro coletas, foram extraídas a partir do software os dados de palavras por minuto, tempo total de leitura e desempenho na tarefa de compreensão. Os dados obtidos foram inseridos em planilha de Excel para conferência e processamento. Foram calculadas medidas de tendência central e de dispersão para cada uma das variáveis estudadas. Para verificar a evolução do desempenho em fluência e compreensão leitora ao longo das testagens foi utilizado o teste de Wilcoxon com nível de significância de 5%. Para análise estatística foi utilizado o software SPSS, versão 21.0.

## RESULTADOS

As Tabelas 1 e 2 apresentam os resultados da fluência e da compreensão de leitura ao longo das quatro testagens. Em relação à fluência de leitura, é possível verificar a evolução desse parâmetro, principalmente nas testagens três e quatro (Tabela 1). Observa-se ainda que na segunda testagem os valores tiveram discretas diminuições, que pode ser devido ao tamanho da amostra. A variabilidade intragrupo é notável, visto os valores altos do desvio padrão e a diferença entre os valores de mínimo e máximo. A Tabela 2 mostra diferenças estatisticamente significantes em todas as comparações realizadas.

**Tabela 1:** Medidas descritivas da fluência e da compreensão de leitura ao longo das quatro testagens

	**FLUÊNCIA**			
	**PPM1 (n=42)**	**PPM2 (n=18)**	**PPM3 (n=43)**	**PPM4 (n=28)**
Média	67	65,06	84,12	91,75
Mediana	69,5	66,5	86	86,5
Desvio padrão	25,9	26,47	26,46	29,1
Mínimo	22	26	37	35
Máximo	123	114	134	139
	**COMPREENSÃO**			
	**Literal1 (n=40)**	**Literal2 (n=19)**	**Literal3 (n=33)**	**Literal4 (n=35)**
Média	3,85	4	4,55	4,54
Mediana	4	5	5	5
Desvio padrão	1,25	1,37	0,87	0,74
Mínimo	1	1	1	3
Máximo	5	5	5	5
	**Inferencial1 (n=40)**	**Inferencial2 (n=19)**	**Inferencial3 (n=33)**	**Inferencial4 (n=35)**
Média	3,95	3,58	4,12	3,8
Mediana	4	4	4	4
Desvio padrão	1,06	1,17	0,96	1,18
Mínimo	1	1	0	0
Máximo	5	5	5	5
	**Total1 (n=40)**	**Total2 (n=19)**	**Total3 (n=33)**	**Total4 (n=35)**
Média	7,8	7,58	8,67	8,37
Mediana	8	8	9	9
Desvio padrão	1,9	2,27	1,63	1,73
Mínimo	2	3	1	3
Máximo	10	10	10	10

**Legenda:** PPM= palavras por minuto

**Tabela 2:** Resultado do teste de Wilcoxon para a comparação da fluência e da velocidade de leitura ao longo das testagens

		**TESTAGENS**					
		**1ª x 2ª**	**1ª x 3ª**	**1ª x 4ª**	**2ª x 3ª**	**2ª x 4ª**	**3ª x 4ª**
PPM	p-valor	0,002*	<0,001*	<0,001*	0,003*	0,008*	0,001*
Compreensão literal	p-valor	0,885	0,009*	0,007*	0,443	0,131	0,608
Compreensão inferencial	p-valor	0,317	0,206	0,774	0,257	0,317	0,206
Compreensão Total	p-valor	0,317	0,013*	0,05*	0,268	0,279	0,917

* p<0,05

**Legenda:** PPM= palavras por minuto; teste de Wilcoxon

Quanto ao desempenho das crianças nas questões literais do questionário aplicado após a leitura do texto, na Tabela 1 é possível observar a evolução desse fator principalmente nas testagens três e quatro. Houve evolução estatisticamente significante quando há comparação da primeira testagem com a terceira e quarta testagens (Tabela 2).

Não se observou evolução no desempenho das crianças nas questões inferenciais do questionário, sendo notável certa dificuldade se comparado a questões literais (Tabela 1). Há uma melhora na testagem três, entretanto essa melhora não foi significativa (Tabela 2).

O desempenho total em compreensão de leitura nas quatro testagens variou significativamente a cada testagem havendo evolução ao se compará-las, o que é esperado pelo bom desempenho nas questões literais (Tabela 1). Observa-se ainda que há uma queda de desempenho da primeira para a segunda testagem, assim como da terceira para a quarta testagem. A Tabela 2 mostra que há aumento estatisticamente significante quando se compara a primeira com a terceira e a quarta testagens.

## DISCUSSÃO

O presente estudo buscou avaliar se a fluência e a compreensão leitora variam ao longo do 3^o^ ano do Ensino Fundamental. Os achados do estudo mostram que há evolução de fluência e da compreensão de leitura global ao longo do 3º ano escolar do Ensino Fundamental, as quais foram medidas de forma objetiva e semiautomática por meio eletrônico. A avaliação da fluência de leitura e compreensão foi realizada seguindo arcabouço teórico já solidificado na área^(1,4,8,19-21)^ e os resultados mostraram evolução gradual objetiva mensurável (Tabelas 1 e 2). Tais achados nos permitem ressaltar a importância da inclusão do monitoramento de fluência e de compreensão de leitura ao longo do ano escolar como indicador de desempenho. Com os resultados obtidos pelo monitoramento, a equipe pedagógica poderá delinear os ajustes necessários no planejamento para o aprimoramento do desempenho dos estudantes tanto na fluência quanto na compreensão leitora.

De acordo com os resultados, a fluência leitora também sofre variação em um mesmo ano escolar, sendo considerado um indicador prático de qualidade, não só para uma avaliação precisa do desempenho dos escolares, mas também para acompanhá-los ao longo do ano e no avanço da seriação escolar, o que corrobora a literatura^(20,21)^.

Alguns fatores podem promover uma melhor fluência leitora, como palavras mais curtas, que tendem a ser mais facilmente decodificadas, assim como as de estrutura sintática mais simples^(12)^ e o número de pausas durante a leitura do texto^(4)^. Estudo realizado com 97 escolares do 3º, 4º e 5º ano^(4)^ apontou relação entre o número de pausas e a taxa de leitura para estudantes do 3º ano. Estes estudantes apresentaram mais pausas, dificuldades na decodificação, leitura mais lenta e dificuldade na compreensão. Dados estes que levam a crer que uma possível consolidação da leitura no 3º ano vai depender do processo de aprendizagem de cada escolar. Assim, esta análise se torna relevante pois na nossa amostra a fluência leitora no 3º ano ainda não estava estabelecida, mas se encontrava em processo de evolução do desempenho.

Tendo em vista os resultados encontrados no presente estudo, no que se refere à velocidade de leitura ao longo das quatro testagens, observou-se que apenas na última avaliação a média foi compatível com o preconizado pela PNA (90 palavras por minuto), que por enquanto são os únicos valores referidos nas políticas públicas nacionais. Tal meta foi alcançada apenas ao final do ano, após as experiências de leitura nas diversas disciplinas, que estimularam as habilidades de literacia comuns em diferentes tarefas. Cerca de 50% dos escolares não atingiram a meta, o que confirma a necessidade de se ter mecanismos para monitoramento da fluência de leitura nas escolas não somente para avaliação pontual, mas também para verificar a fluência de leitura durante o ano para que possam detectar o que está acontecendo dentro dos parâmetros de fluência de leitura e assim traçar estratégias.

Em relação à compreensão, a partir das questões literais não foi observada evolução dos escolares a cada bimestre, houve melhora quando comparada a primeira com a terceira e quarta testagem. Quanto à compreensão inferencial, não se observou evolução ao longo do ano letivo. Em estudo com objetivo de avaliar a compreensão do texto em crianças do segundo e terceiro ano do ensino fundamental foi aplicado um questionário a partir da leitura do texto "A Coisa", mesmo instrumento utilizado no presente estudo. As questões literais são apontadas como questões relacionadas à memória, chamando-as de memória para eventos^(13)^. Nos resultados, o desempenho para questões literais se mostrou um pouco maior que questões inferenciais, o que condiz com nossos achados.

Cunha e Capellini^(17)^ também constataram em seus resultados desempenho inferior quanto a perguntas inferenciais, ocorrendo maior dificuldade por parte dos escolares visto que muitas vezes não há conhecimentos prévios suficientes para que haja vinculação de ideias, o que corrobora também o presente estudo. Tais achados reforçam a necessidade de um trabalho sistematizado e explícito nas séries iniciais do EF com foco na compreensão leitora, além das outras habilidades apontadas no NRP, principalmente no vocabulário^(22)^. Leitores mais informados sobre um tópico podem atrair mais inferências e criar um raciocínio mais rico e representações mais coerentes, permitindo, assim, uma melhor compreensão, identificação de significados e a habilidade de relacionar o texto apresentado a partir de conhecimentos prévios^(23)^. A maior dificuldade das crianças nas questões inferenciais isoladamente não justifica como foi o desenvolvimento desse parâmetro. Análise do currículo escolar e um estudo aprofundado das discussões textuais realizadas em sala podem auxiliar no entendimento dessa questão.

Outras hipóteses foram levantadas a respeito das dificuldades de compreensão, que podem ser devido a fatores extrínsecos ou intrínsecos. Em relação aos fatores intrínsecos, destacam-se as habilidades linguísticas, relacionadas à capacidade de realizar inferências, e habilidades cognitivas, relacionadas ao conhecimento prévio. Já os fatores extrínsecos podem se dar a aspectos socioeconômicos, saúde debilitada, faltas frequentes e um ambiente escolar inadequado^(24)^. A escola onde foi realizada a pesquisa localiza-se em região de maior vulnerabilidade social, o que pode ter interferido no desempenho das crianças. O relatório de 2018 da Codeplan (Secretaria de fazenda, planejamento, orçamento e gestão do Distrito Federal) aponta que a escola na qual a pesquisa foi realizada é localizada na região administrativa Samambaia do Distrito Federal, que faz parte da unidade de planejamento oeste junto com Taguatinga, Sol Nascente, Ceilândia e Brazlândia, região mais populoso do DF. A maioria das pessoas que se declaram estudantes entre 4 e 24 anos frequentam escola pública. Na faixa etária estudada, classificada entre 6 e 14 anos no relatório do GDF, 97% frequentam escola, sendo que esse valor diminui com o avanço da idade (86% entre 15 e 17 anos). Da população com 25 anos ou mais, 35,7% têm ensino médio completo, 22,7% fundamental incompleto e 4,7% sem escolaridade. A taxa de participação para Samambaia era de 63,7% no período de coleta de dados (2018). Destaca-se que 35,5% da população entre 18 e 29 anos não trabalhavm nem estudavam. Apenas 62,8% dos trabalhadores informaram ter carteira assinada. 70,9% da população tem rendimento bruto de até 2 salários mínimos^(25)^.

Outro achado desta pesquisa diz respeito à alta variabilidade intragrupo (Tabela 1). O valor alto de desvio padrão nos padrões de fluência de leitura foram também verificados em outros estudos sobre o tema no Brasil^(26,27)^. Tais achados mostram que nas séries iniciais da alfabetização, os escolares se desenvolvem com ritmo diferente, sendo importante para os educadores compreenderem as diferenças sem deixar de investir na fluência leitora.

Nesse sentido, desenvolver conhecimento é a melhor maneira de melhorar a compreensão da leitura^(14)^. Esta construção deve ocorrer nas primeiras séries escolares, ouvindo, conversando, com atividades, bem como através da leitura propriamente dita.

No presente estudo optou-se por monitorar a fluência e a compreensão leitora a cada bimestre, coincidindo com as demais avaliações realizadas pelos professores, enquanto estudos internacionais realizam as avaliações a cada quatro meses^(20,21)^. Os resultados encontrados, principalmente no que se refere à compreensão leitora, demonstram que três avaliações por ano são suficientes. Dessa forma, sugere-se que o Fonoaudiólogo Educacional e a equipe pedagógica façam as avaliações no início, no meio e no final do ano letivo com instrumentos validados.

As limitações do estudo foram o tamanho da amostra e a inviabilidade de todos os escolares serem testados em todas as etapas devido à falta do aluno no dia da coleta, ou pelo mesmo se recusar a ler quando foi solicitada a leitura em voz alta, ou pela não liberação por parte dos professores para sair da sala durante a aula. A não liberação ocorria também devido a eventos que ocorriam constantemente na escola, ou até mesmo cancelamento das aulas, sem que as pesquisadoras fossem informadas com antecedência.

Segundo Arnesen et al.^(20)^, é esperado que haja essa perda de dados por faltas do aluno já que imprevistos são esperados durante um estudo longitudinal prospectivo. A falta pode ocorrer não só por parte do aluno, como também do professor, por questões de saúde, podendo ocorrer também transferência dos alunos para outra escola, o que também foi constatado.

Quanto ao uso do mesmo texto ter sido utilizado nas 4 avaliações desta pesquisa, isso não se mostrou um problema ou fator limitante já que observamos uma dificuldade persistente em alguns estudantes. Além disso, de acordo com uma meta-análise, a melhora da fluência só é possível com mais de 4 repetições de um mesmo texto momentos antes da avaliação^(8,28)^.

## CONCLUSÃO

A fluência e a compreensão leitora variaram ao longo do ano letivo em crianças do 3º ano do EF, podendo ser usados como indicadores de desempenho de leitura nessa população. A partir dos resultados encontrados e de estudos internacionais, sugere-se um rastreio no início do ano letivo e monitoramento no meio e no final, o que permitirá a intervenção precoce, diminuindo as taxas de insucesso. Esse monitoramento se torna mais preciso e palpável por meio da ferramenta *LEPIC*, pois além de ser fácil de utilizar, a avaliação através do software é rápida e automática, fornecendo dados individuais e coletivos, permitindo a identificação dos alunos que necessitam mudanças na estratégia de ensino.

O uso do *software LEPIC* mostrou-se ser uma ferramenta válida e exequível para o rastreio e monitoramento. Além disso, pode ser utilizado por qualquer pessoa, inclusive os próprios professores, desde que previamente treinados.
